# Rapid and automated screening of carbapenemase- and ESBL-producing Gram-negative bacteria from rectal swabs using chromogenic agar media and the ScanStation device

**DOI:** 10.1128/spectrum.02723-23

**Published:** 2023-09-29

**Authors:** Julien Peyroux, Iyad Almahmoudh, Emelise Prebe-Coquerel, Thomas Girard, Max Maurin, Yvan Caspar

**Affiliations:** 1 Laboratory of Bacteriology, Grenoble Alpes University Hospital, Grenoble, France; 2 Univ. Grenoble Alpes, CNRS, Grenoble INP, TIMC, Grenoble, France; 3 Univ. Grenoble Alpes, LIG, CNRS Grenoble, Grenoble, France; 4 Univ. Grenoble Alpes, CEA, CNRS, IBS, Grenoble, France; Ann & Robert H. Lurie Children's Hospital of Chicago, Chicago, Illinois, USA

**Keywords:** bacterial colonies, detection, digital imaging, multidrug-resistant bacteria, real-time screening, visual observation

## Abstract

The ScanSation 100 device (Interscience, France) is an incubator allowing real-time detection of bacterial colony growth by frequently imaging agar plates over time, counting CFU, and detecting colony color. This study evaluated its performance for the early detection of carbapenemase-producing bacteria (CPB) and extended-spectrum β-Lactamase-producing bacteria (ESBL-PB) from rectal swabs inoculated on CHROMagar mSuperCARBA and ESBL media, respectively. Rectal screening ESwabs collected from patients admitted to Grenoble University Hospital between January and June 2021 were analyzed. After inoculation, chromogenic media were incubated for 24 h in the automaton, with image acquisition every 30 min. ScanStation results were compared to visual observations of the plates after 24 h of incubation. In total, 501 rectal swabs were tested. ScanStation showed 100% positive percent agreement (PPA) for the detection of CPB and ESBL-PB, whereas the PPA of color categorization ranged between 45% and 100%. Negative percent agreement (NPA) ranged between 70% and 98%. Negative predictive values (NPVs) were 100% for both bacterial groups, whereas positive predictive values (PPVs) were 70.3% for CPB and 81.0% for ESBL-PB. Importantly, real-time screening allowed detection of the first suspected colony within 10–14 h of growth, on average, whereas visual observation is usually only performed once a day after 18–24 h of incubation. Our study demonstrates the potential use of early images to accelerate the detection of CPB and ESBL-PB and implement effective and timely infection control measures.

**IMPORTANCE:**

The ScanStation 100 device is an incubator able to follow the real-time growth of bacterial colonies on agar plates through digital imaging, allowing users to sort plates according to the presence or absence of colonies, and to distinguish their color using four numeric color filters. Real-time screening shows that first colony detection is possible much earlier (after 10–14 h of growth, on average), whereas visual observation is usually performed only once a day after 18–24 h of incubation. The ScanStation device, combined with chromogenic agar media, is an efficient automated screening method to accelerate the detection of Gram-negative multidrug-resistant bacteria in laboratories that do not have access to larger laboratory automation systems. Our study shows that setting the image acquisition to one or two early images may allow for the detection of positive samples that were inoculated in the morning, by the end of the working day.

## INTRODUCTION

Many advances have been achieved in recent years in the automation of bacteriology laboratories, allowing for drastic decreases in turnaround time and shortening the time to results for bacterial identification and antimicrobial resistance assessments (1). Culture methods can sometimes be replaced by faster molecular assays, but their higher cost limits high volume analysis (2). Consequently, bacterial culture currently remains the gold standard in most laboratories, in particular for the screening of multidrug-resistant (MDR) bacteria in humans (3).

Every day, many patients are screened for extended-spectrum β-Lactamase-producing bacteria (ESBL-PB) and carbapenemase-producing bacteria (CPB) carriage from rectal swabs (4). They are among the WHO’s priority pathogens due to their increased prevalence of antibiotic resistance in recent decades (5). Systematic screening of inpatients, especially those who have traveled abroad, those who will undergo invasive surgeries, or those who are admitted to the Intensive Care Unit (ICU), is necessary to avert possible hospital outbreaks that can be prevented by rapid infection control measures (6). This screening relies on culturing on selective agar media, followed by: (i) rapid species identification by MALDI-TOF mass spectrometry, and (ii) confirmation of resistance phenotypes by various assays, including rapid immunochromatographic, colorimetric, or molecular assays, or other time-consuming antimicrobial susceptibility testing methods (7 – 9).

Using culture methods, the main steps of extending the detection of MDR bacteria are the growth delay on the selective medium, which is dependent on both the bacterial species and the regularity with which plates are read. Usually, the plates are read only once a day, based on reading times recommended by the manufacturers of selective agar media. Accelerating bacterial detection through real-time analysis of the growing colonies at a low cost could shorten the time-to-result, while remaining affordable for mass screenings.

The ScanStation 100 device (Interscience, France) is an incubator able to follow the real-time growth of bacterial colonies on agar plates over time by digitally imaging the plates at regular intervals. Its software includes a bacterial colony counter and up to four numeric color filters that allow sorting of colonies according to their color. When combined with the use of chromogenic agar plates, it could allow both color-based detection and categorization of the growing colonies. This process has potential for rapid automated and low-cost screening of MDR bacteria on selective chromogenic media, allowing users to more rapidly conduct confirmation assays and implement infection control measures. The work presented here aims to evaluate the performance of the ScanStation 100 device when combined with the use of CHROMagar mSuperCARBA or ESBL chromogenic selective media for the early detection of CPB or ESBL-PB from rectal swabs compared to visual analysis of the agar plates.

## MATERIALS AND METHODS

### Study design and sample collection

This was a comparative study conducted in the bacteriology laboratory of Grenoble Alpes University Hospital, France. Rectal Eswabs (BD Eswab, Pont de Claix, France) collected from patients over 18 years old and sent to the bacteriology laboratory from January 2021 to June 2021 were included in the analysis. This non-interventional study involving data and samples from human participants was carried out according to the Declaration of Helsinki and current French regulations. The investigator (Yvan Caspar, Pharm.D., Ph.D.) signed a commitment to comply with Reference Methodology No. 004 issued by the French authorities, Commission Nationale de l'Informatique et des Libertés. Participants were all informed and did not object to the study. Written consent for participation was not required for this study, in accordance with national legislation and institutional requirements.

### Automated plating of chromogenic agar plates and analysis with ScanStation automaton

We plated 10 µL of samples on selective CHROMagar mSuperCARBA and ESBL plates for rectal Eswabs using the BD Kiestra lab automaton (InoculA specimen processor, BD Diagnostics, France) and a specific exhaustion pattern (4-quadrant S200 pattern, BD Kiestra). Then, agar plates were inserted into a ScanStation device, incubated at 37°C for 24 hr, with digital imaging of the plates performed every 30 min by the automaton.

Each plate was automatically analyzed by the ScanStation software for colony detection, CFU counts, and color classification of the colonies based on specific color filter settings provided by Interscience for each chromogenic agar plate used in this study, following their specifications. On CHROMagar mSuperCARBA plates, *Escherichia coli* are colored dark pink, while other coliforms (*Klebsiella* sp.*, Enterobacter* sp., *Citrobacter* sp., and *Serratia* sp.) are metallic blue, and other carbapenemase-producing organisms (CPOs) are colorless. On CHROMagar ESBL plates, *E. coli* are dark pink, whereas *Klebsiella*, *Enterobacter*, and *Citrobacter* (KEC) are metallic blue, *Proteus* spp. are brown, *Acinetobacter* spp. are cream, *Pseudomonas* spp. are translucent, and *Stenotrophomonas* spp. are colorless. As the ScanStation device is only able to possess four different color filters, while the CHROMAgar ESBL plate has a range of six different colors, the “cream,” “translucent,” and “colorless” categories were regrouped into one color filter corresponding to a “colorless” category.

**Fig 1:**
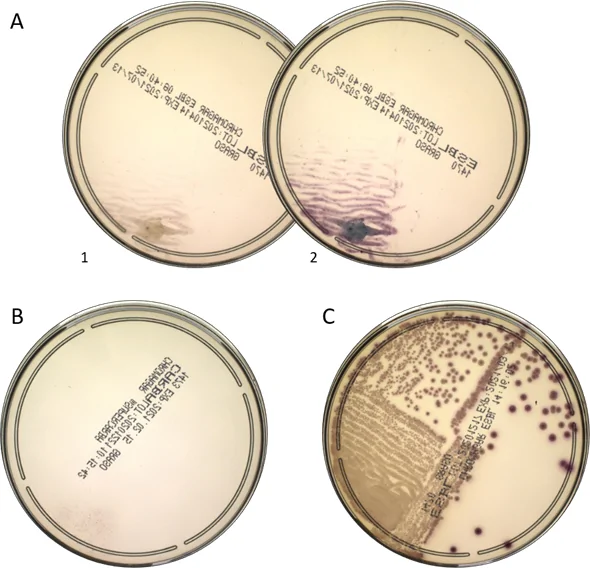
Examples of false positive and false negative results observed during the ScanStation analysis. (A) Artifacts caused by rectal sample seeding before any incubation (i) and after 24 h of incubation (ii), causing positive detection because of the coloration of the agar in the absence of colonies. (B) Late-growing colonies appearing after 20 h of incubation. (C) Chromogenic compound exhaustion causing fading of pink colonies, leading to misidentification of the colonies as colorless.

Data on the times of detection of the first colony on a plate, and of 50% and 95% of all the colonies present at the end of incubation, regardless of their color classification, were extracted. Moreover, times of accurate color categorization, corresponding to the moment the device categorizes the colonies within the right color category, were also extracted from the reports for each sample (Fig. S1). ScanStation results were compared to the visual analysis of the agar plate by lab technicians at the end of the ScanStation analysis (24 h), corresponding to the standard visual analysis usually performed. Colony colors, CFU counts (classified in the ranges 0–10, 11–100, 101–300, and over 300) (Table S1), and the confluence of bacterial colonies were noted for each sample. Samples containing very small colonies during visual examination at the end of incubation that were not compatible with MDR bacterial species screened on those agar plates were considered negative for MDR bacterial screening by visual examination (Fig. 1B). In cases of divergent color classification between the technologist and the Scanstation device, a second visual observation was performed by a second technologist. We determined the positive percent agreement (PPA), negative percent agreement (NPA), positive predictive value (PPV), and negative predictive value (NPV) of MDR-bacterial screening by the ScanStation device compared to results obtained by visual analysis of the agar plates.

## RESULTS

In total, 501 rectal swabs were analyzed. Data on the PPA, NPA, PPV, and NPV of real-time detection and color classification of the growing colonies by the ScanStation device compared to visual analysis of the chromogenic plates are presented in Table 1.

**TABLE 1:** Positive and negative percent agreements and predictive values of the screening of carbapenemase-producing or ESBL-producing Gram-negative bacteria using CHROMagar chromogenic media and the ScanStation device compared to visual analysis of the plates^*a*^

	PPA	NPA	PPV	NPV	True positive	True negative	False positive	False negative
CPB								
All	100.00	88.27	70.32	100.00	109	346	46	0
*E. coli*	100.00	78.23	11.67	100.00	14	381	106	0
Coliforms	100.00	94.06	30.95	100.00	13	459	29	0
CPO	77.32	95.30	79.79	94.59	75	385	19	22
ESBL-PB								
All	100.00	85.94	81.03	100.00	188	269	44	0
*E. coli*	100.00	70.00	35.50	100.00	71	301	129	0
KEC	95.08	98.41	89.23	99.31	58	433	7	3
*Proteus*	100.00	80.04	17.24	100.00	20	385	96	0
APS	45.45	90.33	46.05	90.12	35	393	41	42

^*a*^ PPA: Positive Percent Agreement; NPA: Negative Percent Agreement; PPV: Positive Predictive Value; NPV: Negative Predictive Value; KEC: *Klebsiella*, *Enterobacter*, *Citrobacter*; Coliforms: *Klebsiella*, *Enterobacter*, *Citrobacter*, and *Serratia*; CPB: Carbapenemase Producing Bacteria; ESBL-PB: Extended Spectrum β-Lactamase Producing Bacteria; CPO : other carbapenemase-producing organisms; APS : *Acinetobacter* spp., *Pseudomonas* spp., and *Stenotrophomonas* spp.

Visual observation of CHROMAgar mSuperCARBA plates after 24 h of incubation revealed 109/501 (21.8%) plates positive for MDR bacteria screening: 6 with dark pink colonies evocating *E. coli*, 4 with metallic blue colonies compatible with coliforms, 85 with colorless colonies indicating other Gram-negative bacteria, and 14 samples with mixed colony colors, all requiring further confirmation tests (Table 2; Table S2). Among the 392/501 negative plates (78.2%), 368 plates were empty, and 24 showed late-growing colonies too small to be compatible with CPB. Independent of color classification, ScanStation identified 155/501 positive (30.9%) and 346/501 negative samples (69.1%), without any false negative results. Among the positive samples, 46/155 (29.7%) were false positives, attributed to plate staining or artifacts caused by sample deposition in 17/46 samples (37%) (Fig. 1A), late growing colonies in 17/46 samples (37%), condensation in the cover in 6/46 samples (13%), homogeneity artifacts in the plates in 4/46 samples (9%), and plate edge detection in 2/46 samples (4%).

**TABLE 2:** Correlation table between visual observation and ScanStation results for analysis of carbapenemase-producing bacteria (CPB)^*a*^

CPB			ScanStation					
			Pink	Blue	Colorless	Mix	Negative	Total
Visual observation	Positive	Pink	4	0	0	2	0	6
		Blue	0	1	0	3	0	4
		Colorless	18	3	16	48	0	85
		Mix	0	0	0	14	0	14
	Negative	Late growing	9	1	3	4	7	24
		Empty	18	2	5	4	339	368
		Total	49	7	24	75	346	501

^*a*^ Positive results are differentiated by the color of the colonies. Negative results by visual analysis regroups empty plate results and detection of small late growing colonies not compatible with the MDR bacteria screened on such media. Mix: at least two different color categories were seen on the agar plate (see Tables S2 and S3 for detailed analysis).

After color analysis, no false negative results were observed containing pink or metallic blue colonies. However, ScanStation analysis identified pink colonies in 120 samples, although only 14/120 (12%) were true positives. The false positive results included 34/106 negative plates (32%; 21 empty plates and 13 with late-growing colonies), and misidentification of colorless colonies as pink in 69/106 samples (65%) and as metallic blue in 3/106 samples (3%). Similarly, ScanStation’s detection of metallic blue colonies identified 13/42 (31%) true positive samples. The 29/42 (69%) false positives were due to misidentification of other colorless colonies (21) and pink (1) as coliforms and of positive detection of seven negative plates (six empty plates and one with late-growing colonies). Finally, the detection of colorless colonies resulted in 75/94 (80%) true positive results. Misdetection of negative plates was observed in 15 samples (8 empty plates and 7 with late-growing colonies), while misidentification of pink colonies occurred in four samples. Moreover, 22 false negative results were caused by the misidentification of colorless colonies as pink in 18 samples or as metallic blue in four samples.

The 501 fecal samples were also screened for ESBL-PB. As color classification by ScanStation is limited to four categories, we only distinguished pink colonies compatible with *E. coli*, metallic blue colonies indicative of KEC species, brown colonies representing *Proteus* species, and colorless colonies as potential *Acinetobater*, *Pseudomonas*, and/or *Stenotrophomonas* (APS) species. After 24 h of incubation, visual analysis of the plates showed 188/501 positive samples (37.5%): 48 showed pink colonies, 36 metallic blue colonies, 8 brown colonies, 57 colorless colonies, and 39 mixed colony colors (Table 3; Table S2). The other 313/501 samples (62.5%) were negative. Independent of color classification, 232/501 plates (46.3%) were identified as positive by ScanStation analysis. No false negative result was observed, but 44/232 positive results were false positives (19%). They were related to plate staining or artifacts caused by sample deposition in 23/44 samples (52%), detection of late-growing colonies in 9/44 samples (21%), plate edge detection in 6/44 samples (14%), condensation detection in 5/44 samples (11%), and one agar plate with defects (2%).

**TABLE 3:** Correlation table between visual observation and ScanStation results for analysis of extended-spectrum β-Lactamase-producing bacteria (ESBL-PB)^*a*^

ESBL-PB			ScanStation						
			Pink	Blue	Brown	Colorless	Mix	Negative	Total
Visual observation	Positive	Pink	29	0	0	0	19	0	48
		Blue	2	5	0	0	29	0	36
		Brown	0	0	2	0	6	0	8
		Colorless	17	0	7	2	31	0	57
		Mix	0	0	0	0	39	0	39
	Negative	Late growing	6	0	0	0	3	6	15
		Empty	27	0	1	2	5	263	298
		Total	81	5	10	4	132	269	501

^*a*^ Positive results are differentiated by the color of the colonies. Negative results by visual analysis regroups empty plate results and detection of small, late growing colonies not compatible with the MDR bacteria screened on such media. Mix: at least two different color categories were seen on the agar plate (see Tables S2 and S3 for detailed analysis).

After color analysis, 200 samples were categorized by ScanStation as showing pink colonies, but 129/200 (64.5%) were false positive results (32 samples with metallic blue colonies at visual observation, 6 with brown colonies, 51 with colorless colonies, and 40 negative plates). Metallic blue colony detection by the ScanStation device resulted in 58/65 (89.2%) true positive and 3/436 (0.7%) false negative results (misidentification of metallic blue colonies as pink for all three samples). The seven false positive results resulted from erroneous identification as metallic blue of pink colonies in two samples, colorless colonies in three samples, and false detection in two negative plates. ScanStation detection of brown colonies resulted in 20/116 (17%) true positive and 96/116 (83%) false positive results related to misidentification as brown of pink colonies in 27 samples, of metallic blue colonies in 18 samples, of colorless colonies in 44 samples, and erroneous detection in seven negative plates. Finally, detection of colorless colonies provided 35/76 true positive (46%) and 41/76 false positive results (54%). The 41 false positive detections were due to misidentification as colorless pink colonies in 10 samples, metallic blue colonies in 23 samples, brown colonies in two samples, and incorrect detection of six negative samples. The 42 false negative results were due to colonies being wrongly categorized as pink in 26 samples, metallic blue in one sample, and brown in 15 samples.

Regarding detection delays, the times of detection of the first colony, of 50% and of 95% of all the colonies present after 24 h of growth, and the time of accurate ScanStation color categorization of the colonies from each agar plate are presented in Table 4. On the mSuperCARBA medium, first colony detection was obtained following 13 h 19 min of incubation, with an average delay between detection and correct color categorization of 1 h 59 min. Finally, putative ESBL-PB were detected within 10 h 23 min, with an average delay for correct color categorization of 2 h 15 min.

**TABLE 4:** Average time to results for the detection and color classification of CPB and ESBL-PB by the ScanStation automaton

		Mean time to result (range)	
		CPB	ESBL-PB
First colony	Detection	13h19 (5h – 24h)	10h23 (4h30 – 24 h)
	Color classification	15h18 (5h – 24h)	12h38 (5h – 24 h)
	Delay	1h59 (0h – 18h)	2h15 (0 h - 15h30)
50% colonies	Detection	16h34 (5h – 24h)	13h22 (5h – 24h)
	Color classification	17h32 (6h – 24h)	15h22 (5h – 24h)
	Delay	0h58 (0h – 9h)	2h00 (0h – 12h)
95% colonies	Detection	18h41 (5h – 24h)	16h16 (5h – 24h)
	Color classification	19h29 (5h – 24h)	18h12 (5h – 24h)
	Delay	0h48 (0h – 8h)	1h56 (0h – 13h)

## DISCUSSION

As shortening the time to detect carbapenemase- or ESBL-producing Gram-negative bacteria can have a major impact on limiting cross-transmissions within a healthcare center, any technological improvement that helps reduce the time to produce results may be of interest, as long as it remains affordable for high throughput screening methods. Total lab automation, combined with automated analysis of the plates using digital imaging and artificial intelligence software, has spread in clinical microbiology laboratories in recent years (10, 11). Solutions such as PhenoMATRIX, WASPLab Image Analysis Software, or the BD Urine Culture App have mainly been developed for automated colony count and color analysis on chromogenic agar plates for urine samples, but also for the screening of MDR bacteria (1, 8, 12, 13). Global agreements with standard detection techniques are above 90% (1, 12, 13). A 100% sensitivity comes at the price of a lower specificity (around 80–90%), and these solutions have been demonstrated to reduce the turnaround times for negative results (8, 14).

In this study, we evaluated the performance of a strategy combining the use of chromogenic selective media and an automaton, allowing the early detection of growing colonies by real-time imaging of the plates compared to visual examination and sorting of positive plates. As for most chromogenic screening media, CHROMAgar plates have a high sensitivity with mild specificity (i.e., sensitivity of 96.5–9 7.8% and specificity of 60.7–66.1% for CHROMAgar mSuperCARBA) (15, 16). Detection of growing colonies compatible with the presence of MDR-bacteria by the ScanStation device on such media appears highly reliable for the screening of CPB or ESBL-PB. Indeed, without considering a colony’s color, we observed a 100% PPA compared to visual analysis and a PPV of 70–81%. For comparison, a study performed for the detection of carbapenemase-producing *Enterobacterales* using WASPLab Image Analysis Software also showed a sensitivity of 100% and a specificity of 79.4%, with only 17.8% of samples detected positive showing colonies suggestive of *Enterobacteriaceae* (8).

The NPA was over 85% in our study, showing that the ScanStation device is less effective than visual analysis for the screening of negative samples because of the detection of several artifacts. When considering all of the samples without any growth at visual examination, 12.8% of the empty plates were erroneously classified as positive (11.7% for CPB and 14.1% for ESBL-PB). The main reasons for this issue were plate staining or artifacts related to sample deposition in 44% of the false-positive samples. The composition of some samples appeared to cause a chemical reaction with the chromogenic plate, causing a staining of the plate detected by the automaton without any colonies being present (Fig. 1A). Interestingly, in the previous study using WASPLab Image Analysis Software, false positive results given by the software were also largely due to agar discoloration caused by the specimen matrix despite the lack of bacterial growth (8). Other false positive detections were caused by late-growing small colonies (29%), condensation in the cover (12%), agar plate edge detection as colonies (9%), or small defects in agar plate causing artifacts (6%).

The NPV of the automated screening was excellent for all screenings (100% for CPB and ESBL-PB), confirming the potential use of this approach to automatically eliminate the visual reading of plates categorized as negative by the automaton. This strategy could have helped identify and automatically discard negative plates, saved technical time required for these actions, and rapidly provided negative results to stop infection control measures earlier for 54% and 69% of the samples inoculated on CHROMagar ESBL or mSuperCARBA chromogenic plates, respectively, as already confirmed in other studies (8). As expected, PPV was lower (70.3% for CPB and 81.0% for ESBL-PB) because of the positive detection of artifacts, justifying the need for the second step of visual control of the plates detected as positive by the automaton. This could then allow 19–30% of plates detected as positive by ScanStation device to be quickly discarded because of the presence of artifacts or slow-growing bacteria. As a result, further identification of the bacteria by MALDI-TOF mass spectrometry and processing of confirmatory tests for MDR would only be performed on a reduced number of positive samples after visual validation.

Regarding color detection on CHROMagar mSuperCARBA and ESBL chromogenic media, it seems that the ScanStation device has a reliability to identify blue colonies but has difficulties discriminating pink colonies from brown and colorless ones. Detection of metallic blue colonies showed high PPA, NPA, and NPV (of 100%, 94.1%, and 100% on the mSuperCARBA medium, and 95.1%, 98.4%, and 100% on the ESBL medium, respectively). However, detection of pink colonies had a high PPA and NPV (100% on both chromogenic media) but a lower NPA and very weak PPV (78.2% and 11.7% on the mSuperCARBA medium; 70.0% and 35.5% on the ESBL medium, respectively). Indeed, a significant proportion of false positive color classification by the ScanStation device on those media was due to the misidentification of colorless bacteria as pink, pointing toward *E. coli* (86.7% and 60%, respectively), or as brown, pointing toward *Proteus* (35.3% on the CHROMAgar ESBL medium). Colonies initially grow colorless and can become slightly pink or brownish while aging, leading to false positive results. The difference between detection time and the time to accurate color categorization also highlights the delay in color development on chromogenic agar media, which is a slow process (Fig. S1). However, incorrect color categorization by the ScanStation, leading to the misidentification of suspected species, would easily and rapidly be corrected by MALDI-TOF identification before communication of the results.

Incorrect color categorization can also be correlated with the confluence of the colonies. Among the 225 cases of incorrect color categorization detected in all samples, 97/225 (43%) plates exhibited more than 100 colonies, among which 92/97 (95%) showed confluence or clusters of colonies. Indeed, the proximity between colonies can prevent the ScanStation device from detecting and correctly categorizing all of the colonies present on the agar plate. Second, a large number of colonies seems to create variation in their staining, due to exhaustion of the chromogens present in the agar, leading to heterogeneity of the colony color often being less stained than expected (Fig. 1C). All these difficulties in color categorization also explain why more color categories were identified in 25.6% of the plates by the ScanStation device compared to visual observation (17.1% with one supplementary color, 6.7% with two, and 1.8% with three; Table S2).

Several developments in image acquisition or in the ScanStation software could be proposed to reduce false positive detection and improve color categorization. For instance, positive detection of artifacts may be solved by implementing the automatic elimination of artifacts that appear early (within 3 h of incubation) or small colonies that are detected too late during the analysis (small colonies not compatible with the MDR bacterial species screened on the corresponding agar media were generally much smaller than the expected colony size at 24 h). Moreover, the agar is cream-colored, and colonies that have the same color spectrum may be more difficult to identify or categorize. Therefore, potentially switching the pink chromogenic compounds within chromogenic agar to another color may enhance bacterial uptake of these compounds and improve their automated detection.

In most laboratories, bacteria growing on CPB or ESBL-PB screening media are detected after 18–48 h of growth, based on a single daily observation of all agar plates and a last read at the final time recommended by the agar manufacturer (i.e., classically 18–48 h for main CPB or ESBL-PB screening media). Plates are analyzed either manually or using total lab automation and digital imaging within a BD-KIESTRA or a WASP-Lab system, which could also theoretically be programmed to image plates at any interval, including every 30 min if so desired. However, the latter strategy may overload the capacity of the systems and generate more images per day to analyze, either using artificial intelligence software or through visual analysis by technologists. Importantly, imaging of the plates every 30 min by the ScanStation device showed that first colony detection is possible much earlier (after only 10–14 h of growth) in all positive samples, while on average 95% of the colonies are only detected 5–8 h later. These data may be useful to program the laboratory automation system to take one or two early images (i.e., after 10 and 14 h of incubation), irrespective of the system used by the laboratory. This would help to accelerate the detection of MDR Gram-negative bacteria, which are associated with strict infection control measures, especially CPB.

Rapid identification of growing colonies can be obtained within minutes by MALDI-TOF mass spectrometry. Confirmation assays for ESBL or carbapenemase production can also be started earlier, resulting in detection in less than 30 min using rapid chromogenic or colorimetric assays, as presented in the study by Foschi et al. (8). If MDR bacteria are confirmed, full antimicrobial susceptibility testing can be performed, with results available the next day. However, while positive cultures may be worked up earlier, the turnaround time for negative cultures still needs the minimum hours of incubation per the manufacturer’s instruction (e.g., 18 or 48 h depending on the screening media). Negative samples can only be confirmed after completing the incubation period recommended by the manufacturer. Larger studies using automated incubation and screening have to be performed to see if a 24-h incubation period is sufficient to detect all MDR bacteria if samples are processed using lab automation, thus reducing the load on the incubators for media that require 48 h of incubation.

### Conclusion

The ScanStation device, combined with the use of chromogenic media, provides a new rapid automated approach for the screening of CPB and ESBL-PB from rectal swabs in clinical microbiology laboratories that do not have access to large laboratory automation systems or that only have inoculation modules. By allowing frequent imaging of the plates, the ScanStation device accelerates the detection of positive cultures and permits automatic sorting of positive and negative chromogenic plates. Moreover, this study has shown that the first colony detection is possible much earlier, and that setting the image acquisition to one or two early images (after 12 or 14 h) may allow for the detection of positive samples that were inoculated in the morning, by the end of the working day. This would enable infection control measures to be accelerated and help prevent hospital outbreaks by limiting cross-transmissions. Color detection has to be improved on the ScanStation device, but the global sensitivity and specificity of MDR screening will largely remain dependent on the chromogenic agar plates’ performances. Quantification of the pathogens remains approximate, with only the possibility of estimating with logarithmic precision. However, the high negative predictive value of the ScanStation device makes it possible to automatically discard samples without any growth being detected, reducing technical time and allowing for the communication of results as soon as an image has been obtained. Further improvements to the software and color categorization may improve the performance of this automated screening method in the future.

## SUPPLEMENTAL MATERIAL

Supplemental material spectrum.02723-23-s0001.pdf Tables S1 to S3 and Fig. S1.

ASM does not own the copyrights to Supplemental Material that may be linked to, or accessed through, an article. The authors have granted ASM a non-exclusive, world-wide license to publish the Supplemental Material files. Please contact the corresponding author directly for reuse.
