# Supplementary material for: Rapid and automated screening of carbapenemase- and ESBL-producing Gram-negative bacteria from rectal swabs using chromogenic agar media and the ScanStation device
Source: Microbiol Spectr. 2023 Sep 29;11(5):e02723-23. doi: 10.1128/spectrum.02723-23 (PMC10581142; doi:10.1128/spectrum.02723-23)

Supplemental material

TABLE S1: Correlation of sample repartition according to the number of colonies per plate evaluated by visual observation and detected by the ScanStation device. CPB and ESBL-PB analysis have been regrouped. Slow-growing colonies have been included in the total colony count for each plate.

	ScanStation analysis: number of samples (%)						
	CFU counts/plate	No colonies	0 – 10	11 – 100	101 – 300	> 300	Total
Visual observation : Number of samples (%)	No colonies	602 (97,9 %)	4 (0,7 %)	5 (0,8 %)	2 (0,3 %)	2 (0,2 %)	615 (100%)
	0 – 10	59 (30,6 %)	109 (56,5%)	17 (8,8 %)	6 (3,1 %)	1 (1 %)	193 (100%)
	11 – 100	5 (5,9 %)	6 (7,1 %)	59 (69,4%)	33 (21 %)	7 (8,2 %)	85 (100%)
	101 – 300	0 (0 %)	0 (0 %)	11 (27,5 %)	19 (47,5%)	10 (25 %)	40 (100%)
	> 300	0 (0 %)	0 (0 %)	1 (1,5 %)	3 (4,3 %)	65 (94,2 %)	69 (100%)

TABLE S2 Correlation table between visual observation and ScanStation results for rectal swabs with mixed populations (several colony colors) on CHROMagar^{TM} mSuperCARBA medium

ScanStation / Visual observation	Pink	Blue	Colorless	Pink + Blue	Pink + Colorless	Blue + Colorless	Pink + Blue + Colorless
Pink	4				1		
Blue		1		3			
Colorless	18	3	16	1	31	2	15
Pink + Blue							2
Pink + Colorless					4		1
Blue + Colorless						1	5
Pink + Blue + Colorless							1

TABLE S3 Correlation table between visual observations and ScanStation results for rectal swabs with mixed populations (several colony colors) on CHROMagar^{TM} ESBL medium

Scanstation / Visual observation	Pink	Blue	Brown	Colorless	Pink + Blue	Pink + Brown	Blue + Brown	Blue + Colorless	Brown + Colorless	Pink + Blue + Brown	Pink + Blue + Colorless	Pink + Brown + Colorless	Pink + Blue + Brown + Colorless
Pink	29				1	13				1		4	
Blue	2	5			6			2		1	8		12
Brown			2			4			2				
Colorless	17		7	2	1	5			6	1	17		1
Pink + Blue					3					3			6
Pink + Brown						5						1	
Pink + Colorless						3							
Blue + Brown							1						
Blue + Colorless					2	1				3	1		3
Brown + Colorless						2			1			2	
Pink + Blue + Colorless													2

FIG S1 Example of the delay between the detection of potential ESBL-PB colonies and their identification in the correct color. Graph shows the number of colonies detected every 30 minutes for all color categories, with colorless colony corresponding to suspected *Acinetobacter* spp. or *Pseudomonas* spp. or *Stenotrophomonas* spp. (green line), metallic blue colonies corresponding to *Klebsiella* sp., *Enterobacter* sp., or *Citrobacter* sp. (blue line), pink colonies corresponding to *E. coli* (red line), and brown colonies to *Proteus* sp. (yellow line). Images show snapshots of plates taken by the ScanStation device after 9 and 16 h of incubation.

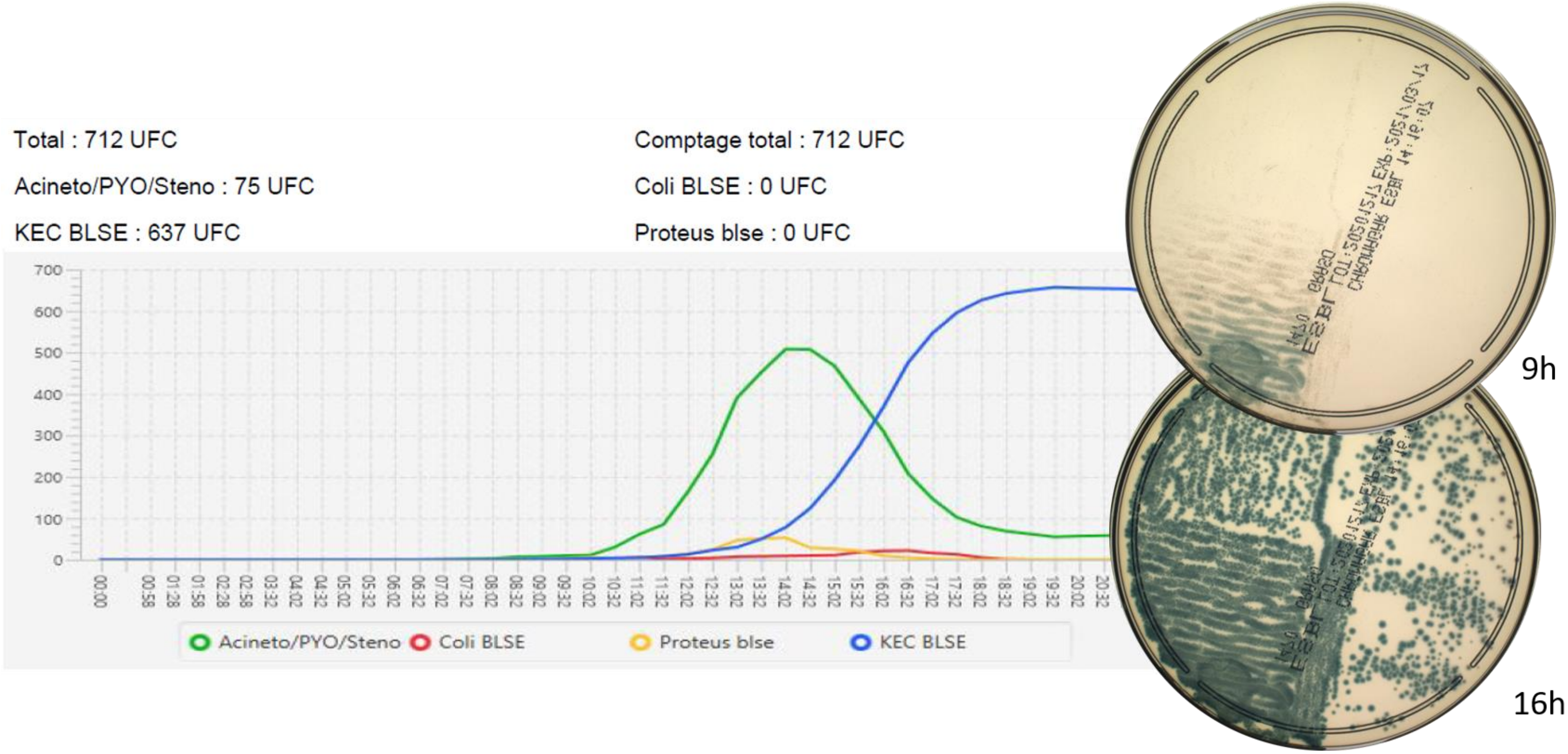

Supplement: Supplemental material — Tables S1 to S3 and Fig. S1. [file spectrum.02723-23-s0001.pdf]
